# Humanized-Aquaporin-4-Expressing Rat Created by Gene-Editing Technology and Its Use to Clarify the Pathology of Neuromyelitis Optica Spectrum Disorder

**DOI:** 10.3390/ijms25158169

**Published:** 2024-07-26

**Authors:** Chihiro Namatame, Yoichiro Abe, Yoshiki Miyasaka, Yoshiki Takai, Yuki Matsumoto, Toshiyuki Takahashi, Tomoji Mashimo, Tatsuro Misu, Kazuo Fujihara, Masato Yasui, Masashi Aoki

**Affiliations:** 1 Department of Neurology, Tohoku University Graduate School of Medicine, Sendai 980-8574, Japan; 2 Department of Pharmacology, Keio University School of Medicine, Tokyo 160-8582, Japan; 3 Laboratory of Reproductive Engineering, Institute of Experimental Animal Sciences, Osaka University Medical School, Suita 565-0871, Japan; 4 Department of Neurology, National Hospital Organization Yonezawa Hospital, Yonezawa 992-1202, Japan; 5 Division of Animal Genetics, Laboratory Animal Research Center, Institute of Medical Science, The University of Tokyo, Tokyo 108-8639, Japan; 6 Department of Multiple Sclerosis & Therapeutics, Fukushima Medical University, Fukushima 960-1295, Japan; 7 Multiple Sclerosis & Neuromyelitis Optica Center, Southern Tohoku Research Institute for Neuroscience, Koriyama 963-8563, Japan

**Keywords:** neuromyelitis optica spectrum disorder, aquaporin-4, NMO-IgG, extracellular domain, humanized-AQP4-expressing rat model, CRISPR/Cas9 genome editing

## Abstract

Conventional rodent neuromyelitis optica spectrum disorder (NMOSD) models using patient-derived immunoglobulin G (IgG) are potentially affected by the differences between the human and rodent aquaporin-4 (AQP4) extracellular domains (ECDs). We hypothesized that the humanization of AQP4 ECDs would make the rodent model lesions closer to human NMOSD pathology. Humanized-AQP4-expressing (hAQP4) rats were generated using genome-editing technology, and the human AQP4-specific monoclonal antibody (mAb) or six patient-derived IgGs were introduced intraperitoneally into hAQP4 rats and wild-type Lewis (WT) rats after immunization with myelin basic protein and complete Freund’s adjuvant. Human AQP4-specific mAb induced astrocyte loss lesions specifically in hAQP4 rats. The patient-derived IgGs also induced NMOSD-like tissue-destructive lesions with AQP4 loss, demyelination, axonal swelling, complement deposition, and marked neutrophil and macrophage/microglia infiltration in hAQP4 rats; however, the difference in AQP4 loss lesion size and infiltrating cells was not significant between hAQP4 and WT rats. The patient-derived IgGs bound to both human and rat AQP4 M23, suggesting their binding to the shared region of human and rat AQP4 ECDs. Anti-AQP4 titers positively correlated with AQP4 loss lesion size and neutrophil and macrophage/microglia infiltration. Considering that patient-derived IgGs vary in binding sites and affinities and some of them may not bind to rodent AQP4, our hAQP4 rat is expected to reproduce NMOSD-like pathology more accurately than WT rats.

## 1. Introduction

Neuromyelitis optica spectrum disorder (NMOSD) is an autoimmune disease of the central nervous system clinically characterized by relapsing optic neuritis and myelitis [1]. Clinically, the acute attacks are often severe and relapsing, causing residual disabilities [2]. Despite acute treatment for NMOSD attacks, such as high-dose intravenous methylprednisolone therapy and/or plasma exchange, most patients experience severe residual symptoms, and only 21.6% showed complete remission [3]. Recently, several high-efficacy treatments for relapse prevention have become available [4,5]; however, some cases still break through those therapies. Therefore, NMOSD animal models are needed to better elucidate the pathogenesis of NMOSD and develop more effective treatments.

NMO-IgG, the autoantibody in NMOSD, was identified as a pathogenic autoantibody against aquaporin-4 (AQP4) [6]. Intraperitoneal injection of NMO-IgGs induced astrocyte injury associated with complement deposition in rat models of experimental autoimmune encephalomyelitis (EAE) immunized with myelin basic protein (MBP) [7,8,9], which established the pathogenicity of anti-AQP4 antibodies. Subsequently, several rodent models have been developed in which anti-AQP4 antibody is injected into the blood or directly into the central nervous system (CNS) [10,11,12,13,14]. However, the lesion caused by the injection of patient-derived IgGs only showed mild perivascular loss of AQP4.

AQP4 is a six-transmembrane water channel protein with extracellular (loop A/C/E) and intracellular (loop B/D) domains, mainly expressed on astrocyte end-feet and ependymal cells in the CNS [15,16,17,18,19]. AQP4 has two dominant isoforms, namely, M1 and M23 [16,20,21,22], that form tetramers in the membrane. M23-AQP4 tetramers form an orthogonal array of particles (OAPs) [23,24,25,26,27]. NMO-IgGs bind to AQP4 extracellular domains (ECDs) [28,29]; however, they are polyclonal [9,30], and the binding sites in AQP4 ECDs differ among patients [31,32,33]. Three amino acids in the rat AQP4 ECDs, namely, Ser62, Asn64, and Thr149, are different from those of human AQP4; this difference can affect the binding capacity of NMO-IgGs in vitro [31,32]. Previously, we reported that three out of five NMOSD patient-derived IgGs failed to recognize mouse AQP4 which contains four different amino acids (Ser62, Asn64, Thr149, and Ala228) in the ECDs compared to human AQP4 [34]. Another study suggested that the binding to A228E mouse AQP4, which has the same amino acid sequence in ECDs as rat AQP4, was weaker in five and unclear in one out of 10 NMOSD patient-derived IgGs than binding to human AQP4 [31]. These findings suggest that some NMOSD patient-derived IgGs might bind less to rodent AQP4 than to human AQP4. However, the relationship between species-associated structural differences of AQP4 and in vivo pathogenicity of NMO-IgGs is unknown. We hypothesized that the difference in these amino acids across species would affect the in vivo pathogenicity of NMO-IgGs and that humanizing the AQP4 ECDs of rat AQP4 might increase the affinity and pathogenicity of patient-derived IgGs to induce severe lesions comparable to the human pathology of NMOSD.

This study aimed to generate the humanized-AQP4-expressing (hAQP4) rat and evaluate its utility as the NMOSD model. We employed our experimental NMOSD model with MBP-EAE [35] and hAQP4 rats to reproduce NMOSD-like lesions by transferring human AQP4-specific antibodies or NMOSD patient-derived IgGs. 

## 2. Results

### 2.1. Human AQP4-Specific Antibody Induced Aastrocyte Injury in the CNS of hAQP4 Rats but Not in WT Rats

**Figure 1:**
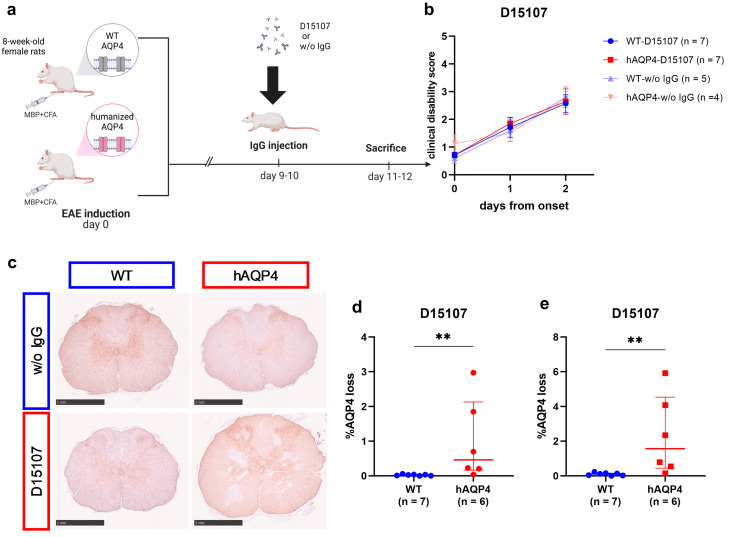
Human AQP4-specific monoclonal antibody D15107 induced astrocyte damage specifically in hAQP4 rats. (**a**) Summary of the experimental protocol. Created with BioRender.com. (**b**) Clinical disability scores of the wild-type Lewis (WT) rats transferred with D15107 (WT-D15107, blue), humanized-aquaporin-4-expressing (hAQP4) rats transferred with D15107 (hAQP4-D15107, red), WT rats without IgG injection (WT-w/o IgG, light blue), and hAQP4 rats without IgG injection (hAQP4-w/o IgG, light red) group on the day of IgG injection (day 0) and after 2 days. Values are mean ± SEM of each group. (**c**–**e**) Sizes of AQP4 loss lesions in the spinal cord induced by human AQP4-specific monoclonal antibody on the pathological examination. The percentage of the AQP4 loss area was calculated for 12 slices per rat by dividing the AQP4 loss area by the whole section area. (**c**) AQP4 staining of the spinal cord sections in each group. Each scale bar = 1 mm. (**d**) Average of 12 slices per rat in the WT-D15107 and hAQP4-D15107 groups. Values are median ± IQR with individual points. (**e**) The average percentage of the AQP4 loss area in the slices with the three largest lesions per rat in the WT-D15107 and hAQP4-D15107 groups. Values are median ± IQR with individual points. Statistical analyses were performed using the Wilcoxon test with GraphPad Prism 8.4.3, and significance is indicated as ** *p* < 0.01.

First, we examined whether human AQP4-specific monoclonal antibody D15107 can induce astrocyte injury in WT and hAQP4 rats (Figure 1a). The clinical disability scores were not significantly different among WT rats without IgG injection (WT-w/o IgG), WT rats transferred with D15107 (WT-D15107), hAQP4 rats without IgG injection (hAQP4-w/o IgG), and hAQP4 rats transferred with D15107 (hAQP4-D15107) (Figure 1b). Histopathological analysis revealed AQP4 loss lesions in the CNS of the hAQP4-D15107 group, whereas the lesions were hardly found in the WT-D15107 group and were not detected in the WT-w/o IgG and hAQP4-w/o IgG groups (Figure 1c–e). The hAQP4-D15107 group had significantly larger lesions than the WT-D15107 group (Figure 1d,e).

### 2.2. NMOSD Patient-Derived IgGs Induced Astrocyte Injury in the CNS of hAQP4 Rats and the Lesion Size Was Dose-Related

Then, we investigated whether NMOSD patient-derived IgGs (Table 1) can induce NMOSD-like lesions in hAQP4 rats and whether the lesion size is IgG dose-related.

**Figure 2:**
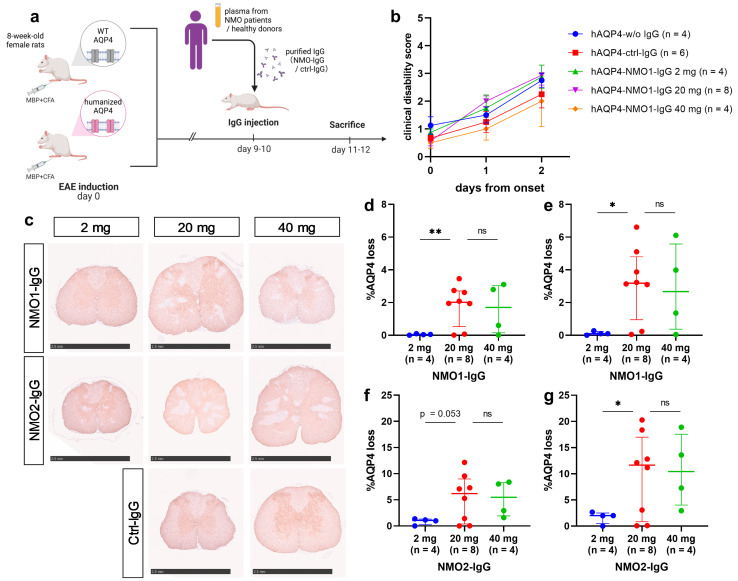
AQP4 loss lesions in hAQP4 rats induced by NMOSD patient-derived IgGs were dose-related. (**a**) Summary of the experimental protocol. Created with BioRender.com. (**b**) Clinical courses of the rats after injection with patient-derived IgGs or ctrl-IgG. Clinical disability scores of the hAQP4 rats without IgG injection (hAQP4-w/o IgG, blue), hAQP4 rats transferred with ctrl-IgG (hAQP4-ctrl-IgG, red), hAQP4 rats transferred with NMO1-IgG (hAQP4-NMO1-IgG) 2 mg (green), hAQP4-NMO1-IgG 20 mg (purple), and hAQP4-NMO1-IgG 40 mg (orange) on the day of IgG injection (day 0) and after 2 days. Values are mean ± SEM of each group. (**c**) AQP4 staining of the spinal cord sections in each group. Each scale bar = 2.5 mm. (**d**–**g**) Size of AQP4 loss lesions in the spinal cord induced by patient-derived IgGs on the pathological examination. The percentage of AQP4 loss area was calculated in 12 slices per rat by dividing the AQP4 loss area by the whole section area. (**d**,**f**) Average percentage of the AQP4 loss area in 12 slices per rat (**d**) in the hAQP4-NMO1-IgG 2 mg, 20 mg, and 40 mg groups and (**f**) in the hAQP4 rats transferred with NMO2-IgG (hAQP4-NMO2-IgG) 2 mg, 20 mg, and 40 mg groups. Values are median ± IQR with individual points. (**e**,**g**) The average percentage of the AQP4 loss area in the slices with the three largest lesions per rat (**e**) in the hAQP4-NMO1-IgG 2 mg, 20 mg, and 40 mg groups and (**g**) in the hAQP4-NMO2-IgG 2 mg, 20 mg, and 40 mg groups. Values are median ± IQR with individual points. Statistical analyses were performed using Dunnett’s T3 test with GraphPad Prism 8.4.3. Significance is indicated as ns: not significant, * *p* < 0.05, ** *p* < 0.01.

**Figure 3:**
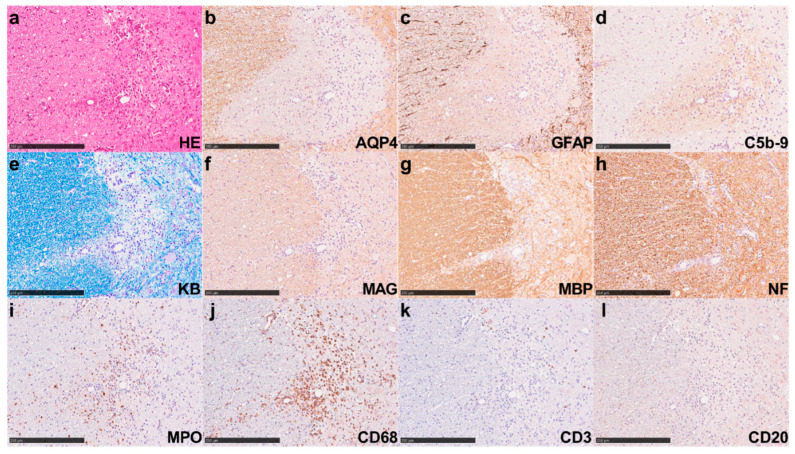
NMOSD patient-derived IgGs induced severe NMOSD-like lesions in hAQP4 rats. Pathological findings in the spinal cord of hAQP4 rats that received 20 mg of NMO2-IgG. (**a**) Hematoxylin and eosin (HE) staining showed vasculocentric tissue-destructive lesions. (**b**,**c**) Immunohistochemistry (IHC) for AQP4 (**b**) and glial fibrillary acidic protein (GFAP) (**c**) revealed complete loss of AQP4 and GFAP, indicating astrocyte damage. (**d**) IHC for C5b-9 showed vasculocentric complement depositions in the lesion. (**e**) Klüver–Barrera (KB) staining indicated obvious demyelination in the center of the lesion. (**f**,**g**) The IHC for myelin-associated glycoprotein (MAG) (**f**) and myelin basic protein (MBP) (**g**) showed a decrease in staining. (**h**) The IHC for neurofilament (NF) showed mild axonal swelling. (**i**,**j**) The IHC for myeloperoxidase (MPO) (**i**), expressed mostly in neutrophils, and CD68 (**j**), expressed mainly in macrophage/microglia, revealed marked infiltration by these cells in the lesion. (**k**,**l**) The IHC for T-cell marker CD3 (**k**) and B-cell marker CD20 (**l**) showed rare infiltration by these cells in the lesion. All these staining procedures were performed using serial sections. All these figures indicate the same lesion. Scale bar = 250 µm.

The symptoms and extent of astrocyte injury in the spinal cords of the hAQP4-w/o IgG, hAQP4 rats transferred with control-IgG (hAQP4-ctrl-IgG), and hAQP4 rats transferred with 2 mg, 20 mg, and 40 mg of NMO1-IgG (hAQP4-NMO1-IgG) were analyzed (Figure 2a). The clinical disability scores of hAQP4 rats after IgG injection were not significantly different among these groups (Figure 2b). However, histological analysis revealed AQP4 loss associated with glial fibrillary acidic protein (GFAP) loss in the CNS of the hAQP4-NMO1-IgG group, whereas no AQP4 loss was observed in the hAQP4-w/o IgG or hAQP4-ctrl-IgG group (Figure 2c). Among the hAQP4-NMO1-IgG groups, the AQP4 loss lesions in the spinal cord sections were larger in the 20 mg and 40 mg groups than in the 2 mg group, and the difference between the 2 mg and 20 mg groups was significant (Figure 2d,e). hAQP4 rats transferred with NMO2-IgG (hAQP4-NMO2-IgG) 2 mg, 20 mg, and 40 mg were also evaluated, and no significant difference in clinical symptoms was found between these groups. Conversely, the 20 mg and 40 mg groups showed larger lesions than the 2 mg group, and the difference between the 2 mg and 20 mg groups was significant in the comparison of the top three sections in terms of AQP4 loss lesion size (Figure 2c,f,g). In the hAQP4 rats that received 20 mg of NMO2-IgG, AQP4 loss lesions were associated with GFAP loss, tissue destruction, activated-complement deposition (Figure 3a–d), demyelination (Figure 3e–g), and axonal swelling (Figure 3h). Marked infiltration by myeloperoxidase (MPO)- and CD68-positive cells was observed in these lesions, whereas few CD3- and CD20-positive cells were detected (Figure 3i–l). Accordingly, we used 20 mg of patient-derived IgGs per rat in subsequent experiments.

### 2.3. NMOSD Patient-Derived IgGs Induced AQP4 Loss Lesions in Both hAQP4 and Wild-Type Rats

**Figure 4:**
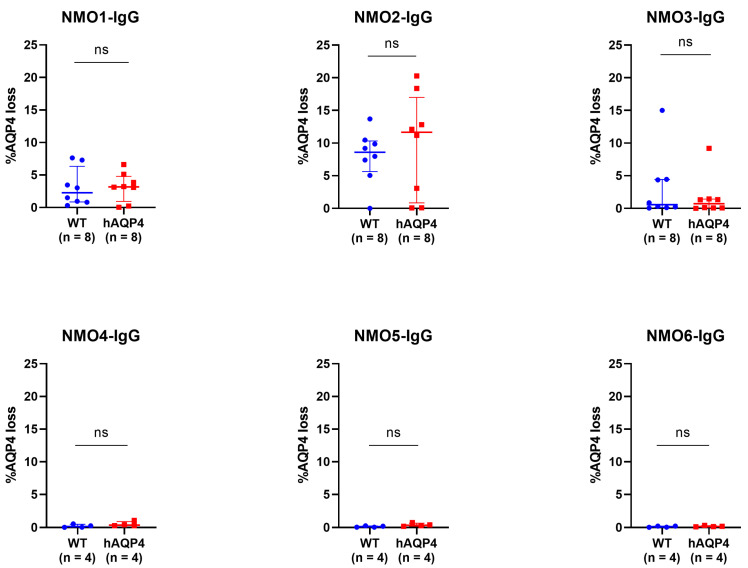
NMOSD patient-derived IgGs induced spinal cord lesions in both WT and hAQP4 rats without significant difference. Percentage of the AQP4 loss area in the spinal cord sections induced by six patient-derived IgGs. The percentage of the AQP4 loss area was calculated in 12 sections per rat by dividing the AQP4 loss area by the whole section area, and the average in the slices with the three largest lesions per rat was plotted on the graph, with median and IQR range indicated. Statistical analyses were performed using the Wilcoxon test for NMO1- to NMO3-IgG and Welch’s *t*-test for NMO4- to NMO6-IgG with GraphPad Prism 8.4.3, and significance is indicated as ns: not significant.

To compare the lesions induced by each patient-derived IgG in WT and hAQP4 rats, experimental NMOSD was induced using 20 mg of IgGs for the remaining NMO3- to NMO6-IgGs. All six patient-derived IgGs induced AQP4 losses in the CNS of both WT and hAQP4 rats without significant difference (Figure 4, upper panel), although only slight lesions were observed in the rats administered low-titer NMO-IgGs (Figure 4, lower panel).

Complement activation and inflammatory cell infiltration in the AQP4 loss lesions were also analyzed. All spinal cord lesions were classified into six types of NMOSD lesions as previously described [36]. Regardless of which patient-derived IgGs were transferred, most lesions in the spinal cords were type 1 lesions (activated complement-induced astrocyte lysis) in both WT and hAQP4 rats. None of the patient-derived IgGs showed a significant difference in the density of infiltrating cells, such as MPO-positive neutrophil, CD68-positive macrophage/microglia, and CD3-positive T-cells in the AQP4 loss lesions, between WT and hAQP4 rats (Figure S1a–c). 

**Figure 5:**
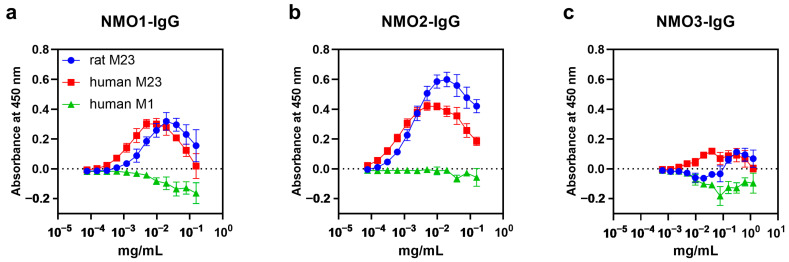
NMOSD patient-derived IgGs bound to both human AQP4 M23 and rat AQP4 M23 but did not bind to human AQP4 M1. (**a**–**c**) Binding of NMO1-IgG (**a**), NMO2-IgG (**b**), and NMO3-IgG (**c**) to rat M23 (blue), human M23 (red), and human M1 (green) expressed in CHO-K1 cells evaluated by enzyme-linked immunosorbent assay (ELISA). Values are mean ± SEM of four independent experiments.

To check the binding affinity of patient-derived IgGs used, a stepwise-diluted affinity assay of patient-derived IgGs to human and rat AQP4 was performed using enzyme-linked immunosorbent assay (ELISA). NMO1-, NMO2-, and NMO3-IgGs bound to human AQP4 M23 and rat AQP4 M23 but not to human AQP4 M1 (Figure 5a–c). The binding of NMO1-, NMO2-, and NMO3-IgG to human AQP4 M23 was detected in lower concentrations than to rat AQP4 M23 (Figure 5a–c). NMO4-, NMO5-, and NMO6-IgGs could not be evaluated because of the limit of detection.

### 2.4. The Clinical Manifestations of the Patients during Acute Attacks Did Not Correlate with the Lesion Distribution of the Experimental NMOSD Model, Whereas the Anti-AQP4 Titers Correlated with the Lesion Size and Infiltrating Cells

Patients’ clinical features and the pathological findings of hAQP4 rats in which patient-derived IgGs were transferred were compared. The expanded disability status scale (EDSS) of the patient at the nadir ranged from 4.0 to 7.5 (Table 1). Among the six patients with NMOSD, three (NMO1, NMO2, and NMO3) poorly recovered clinically after acute treatment, and the others (NMO4, NMO5, and NMO6) recovered well. Despite these variations, the clinical symptoms of the hAQP4 NMOSD model rats were not significantly different between each patient-derived IgG group (Table S1). The clinical phenotypes at the time of attack in each patient varied (Table 1), whereas the distribution of lesions in the rats did not correlate with the lesion distribution in the patients (Table S1). The AQP4 loss lesions in hAQP4 rats were frequently distributed in the cerebrum, pons, medulla, and spinal cord, but less frequently in the optic nerve, midbrain, and cerebellum (Table S1).

**Figure 6:**
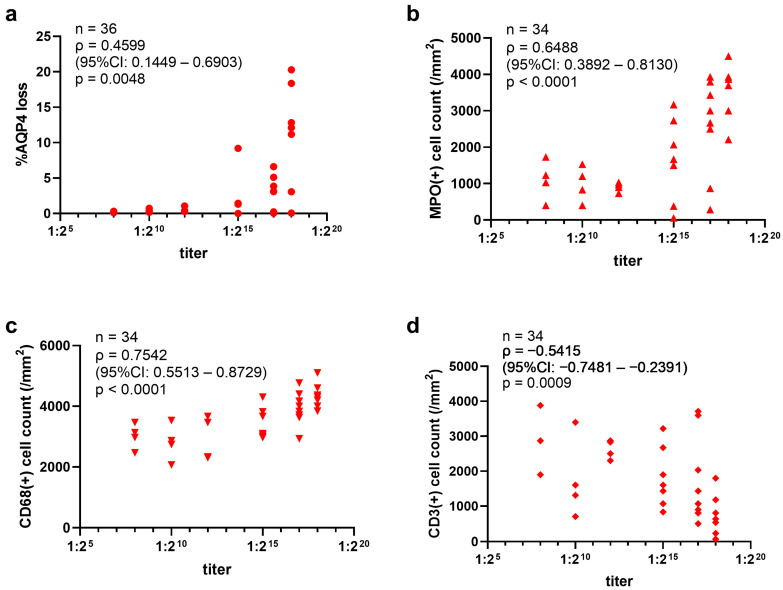
The anti-AQP4 titers of NMOSD patient-derived IgGs correlated with the size of the AQP4 loss lesions and MPO-, CD68-, and CD3-positive cells infiltrating into the lesions in the hAQP4 rat model. (**a**) Relationship between the anti-AQP4 titers of patient-derived IgGs and the size of induced AQP4 loss lesions in the spinal cord of hAQP4 rats. The average percentage of the AQP4 loss area in the slices with the three largest lesions per rat was plotted in the graph. (**b**–**d**) Relationship between the titer of patient-derived IgGs and the density of MPO- (**b**), CD68- (**c**), and CD3-positive (**d**) cells in the AQP4 loss lesions. Statistical analysis was performed using Spearman’s rank correlation coefficient with GraphPad Prism 8.4.3.

On the other hand, the extent of AQP4 loss lesions varied according to the IgGs transferred, and a positive correlation was observed between the lesion size and anti-AQP4 titers of the purified IgG (Figure 6a). In addition, MPO-positive neutrophils and CD68-positive macrophage/microglia infiltrating the AQP4 loss lesions were positively correlated with the titers (Figure 6b,c), whereas CD3-positive T cells were negatively correlated with the titers (Figure 6d). These correlations were found in both hAQP4 and WT rats. Type 1 lesions, characterized by astrocyte loss with C5b-9 deposition, occupied a certain portion of the total lesions regardless of the patient-derived IgG transferred (Table S1). 

## 3. Discussion

### 3.1. Establishment of a New NMOSD Model Using hAQP4 Rats

#### 3.1.1. Reproduction of NMOSD-Like Pathology

A novel NMOSD rat model that expresses AQP4 with humanized ECDs was developed. This rat model can reproduce severe astrocytopathy in the CNS comparable to human NMOSD pathology in the acute phase [36,37,38] after the transfer of human AQP4-specific antibodies or NMOSD patient-derived IgGs. The variations in expressed proteins between animal species often hinder the development of disease models. Recently, gene-editing techniques have advanced considerably, allowing for the precise modification of the target gene to align with specific research purposes. In practice, rodent models with human gene sequences knocked in using gene-editing technology, such as the mouse model of hypertrophic cardiomyopathy [39] and cardiovascular disease [40], have been applied to the research for novel therapies. Regarding research on NMOSD, the differences in AQP4 ECDs between species can affect antibody binding [31,32], and some patient-derived IgGs cannot bind to rodent AQP4 [34], potentially affecting lesion formation in conventional rat models.

We overcame this limitation using gene editing to humanize rat AQP4 ECDs and established a novel NMOSD model that can induce human AQP4-specific antibody binding and subsequent astrocytic lesion formation (Figure 1). Moreover, tissue-destructive lesions with marked astrocyte injury, complement deposition, demyelination, mild axonal swelling, and significant infiltration of MPO-positive cells were found in the CNS of hAQP4 rats in which NMOSD patient-derived IgGs were injected in the blood (Figure 3). These pathological features are comparable to those observed in human NMO pathology [36,37,38]. Additionally, compared to mouse models, the use of rats has several advantages: they have a complement system that can be activated by human IgG [41], thus not requiring the co-injection of human complements; their relatively large size allows for evaluating biomarkers such as GFAP, neurofilament light chain, and cytokines/chemokines in sera and CSF [42]; and they can be investigated using magnetic resonance imaging and somatosensory evoked potentials [14]. As above, the hAQP4 rats can contribute to the comprehensive analyses of NMOSD pathophysiology induced by patient-derived IgGs.

#### 3.1.2. Anti-AQP4 Titer and Lesion Formation

Our pathological analyses revealed that antibody titer evaluated with live cell-based assay correlated with the lesion size in the NMOSD model rats (Figure 6a). The antibody titer reflects several parameters of patient-derived IgGs, such as the amount and affinity of anti-AQP4-IgGs contained in patient-derived IgGs. The dose of the injected IgGs was also related to the lesion size (Figure 2d–g). Although the clinical significance of serum anti-AQP4 titer is controversial, some studies have linked it to the number of involved spinal cord segments [43,44] in the acute phase of NMOSD. Our results support the finding that a high titer of anti-AQP4 antibodies during an acute attack may be a risk factor for the emergence of extensive lesions.

Moreover, the density of lesional neutrophils and macrophage/microglia were positively correlated; however, T cells were negatively correlated with the anti-AQP4 titer of the transferred IgGs (Figure 6b–d). Marked neutrophil infiltration is seen in acute lesions of NMOSD autopsy cases [36,37,38] and in a biopsy of acute AQP4-antibody-positive NMOSD brain lesions [45]. In vitro experiments showed that neutrophils can damage AQP4-expressing CHO cells in the presence of AQP4 antibodies [46]. In a previously reported mouse model, neutrophil elastase inhibitors prevented the expansion of NMOSD lesions [47]. Moreover, a recent study showed that neutrophils release cell-free DNA in patients with NMOSD, which can induce type 1 interferons [48]. These findings indicate that neutrophils exacerbate astrocyte injury during acute NMOSD attacks. Macrophage/microglia infiltration is also one of the pathological features of acute NMOSD lesions [36,37,38]. A previous study suggested that T cells were not required for lesion expansion [49], which is consistent with our results. Thus, our findings suggest that high-titer IgGs quantitatively activate more complements, produce more chemoattractants such as C5a than the low-titer IgGs, and then induce infiltration of numerous neutrophils.

#### 3.1.3. Lesion Distribution

The lesion distribution observed in our model did not correspond to the clinical presentation of each patient (Table S1). Although the factors that determine the lesion site during acute NMOSD attacks remain unclear, previous studies have shown that anti-AQP4 antibody seropositivity alone would not be sufficient to induce the lesions [12] and increased blood–brain barrier (BBB) permeability would also be needed [13]. Some previous studies have noted the potential role of the availability of AQP4 in antigen presentation and CNS antigen-specific T cells [50,51]. A previous study of a rat model using AQP4-specific T cells showed that, in the presence of NMO-IgGs, a low number of T cells induced lesions exclusively in the spinal cords, whereas a higher number of T cells induced brain lesions in addition to affecting the spinal cords [52]. On the other hand, MBP-EAE shows mononuclear cell infiltrations in the cerebrum, pons, and brainstem including the midbrain, and, particularly, the spinal cord [53,54]. The lesion distribution in our rat model resembles that of MBP-EAE, supporting the finding that pathogenic T cells contribute to determining the lesion location. The difference in the factors increasing BBB permeability might have caused the inconsistency in the lesion distribution between NMOSD patients and our rat model.

### 3.2. Influence of Differences between Human and Rat AQP4 ECDs on the NMOSD Model

Human AQP4-specific antibody D15107 recognized human and humanized rat AQP4 equally in the ELISA (Figure S2e) and induced significantly larger astrocyte loss lesions in hAQP4 rats than in WT rats (Figure 1d,e), which is consistent with our hypothesis of the superiority of hAQP4 rats in the induction of NMOSD-like lesions by autoantibodies against human AQP4 ECDs. In this context, the hAQP4 rats could potentially contribute to the development of novel treatments that block the binding of anti-AQP4 antibodies to human AQP4, such as “aquaporumab” [55]. However, in this study, patient-derived IgGs induced NMOSD-like lesions in both WT and hAQP4 rats without significant differences in lesion size, complement activation, and inflammatory cell infiltration (Figure 4 and Figure S1). Although the patient-derived IgGs used had slightly higher affinity to human AQP4, they also bound to rat AQP4 M23 (Figure 5). In addition, the patient-derived IgGs bound minimally to human AQP4 M1 but significantly to human M23 and rat M23 (Figure 5), suggesting that they bind mainly to OAPs in both hAQP4 and WT rats. The binding of anti-AQP4 antibodies to AQP4 OAPs induces complement C1q activation and activates the classical pathway, leading to the formation of a membrane attack complex and eventually astrocyte lysis [56]. Meanwhile, anaphylatoxin C5a, which is produced during the complement activation process, induces neutrophil infiltration, cooperating with granulocyte colony-stimulating factor. In this study, both hAQP4 and WT rats showed the deposition of C5b-9 and marked neutrophil infiltration in the lesions with astrocyte lysis after NMOSD patient-derived IgG injection (Figure 3 and Figure 4). Previous studies have shown that patient-derived IgGs contain polyclonal anti-AQP4 antibodies, and each clone has its own binding site to AQP4 and can mediate complement-dependent cytotoxicity (CDC) and antibody-dependent cellular cytotoxicity [9,30,32]. Anti-AQP4 antibody clones whose binding depends on His151 and Leu154, which are common in human and rat AQP4, showed enhanced CDC compared with clones independent from these amino acids [57], corresponding to our results. On the contrary, some anti-AQP4 antibody clones can even be cytoprotective in the CDC assay [58]; however, such a cytoprotective effect was not detected in this study.

### 3.3. Limitations

This study has some limitations. First, the difference between WT and hAQP4 rats using NMOSD patient-derived IgGs could not be confirmed. In this study, patient-derived IgGs bound to both rat and human AQP4, suggesting that the binding sites of pathogenic anti-AQP4 antibody clones are independent of the difference between rat and human AQP4 ECDs. Although we included only six patients with NMOSD, previous studies have indicated that some NMOSD patient-derived IgGs cannot bind to rodent AQP4 [31,34]; thus, our hAQP4 rats would be appropriate for the evaluation of such IgGs detected in a larger cohort. Second, we used MBP as an immunogen to induce EAE, which is not involved in NMOSD pathology. Because T-cell responses to AQP4 have been reported [59] and AQP4-specific T-cells can cause CNS inflammation in rats [52], our hAQP4 rats could induce pathology closer to human NMOSD by transfer of AQP4-reactive T cells along with NMOSD patient-derived IgGs or direct immunization with AQP4 peptides [60], instead of inducing MBP-EAE. Third, although this model can reproduce acute NMOSD-like pathology (Figure 1c and Figure 2c), clinical exacerbation by IgG injection was hardly detectable in our rat model (Figure 1b and Figure 2b). Since previous NMOSD models without MBP-EAE showed that early clinical exacerbation was induced by patient-derived IgGs [14,61], perhaps MBP-EAE might have masked the symptoms caused by patient-derived IgGs in this study. This limitation could affect the analysis of drug efficacy; however, it could also be solved using the models described above, which eliminate the effects of MBP-EAE. In addition, direct anti-AQP4 antibody injection to the optic nerve [61] and intrathecal patient-derived IgG injection [14] in our hAQP4 rats are expected to further clarify the pathological and clinical implications of NMOSD patient-derived IgGs. Fourth, whether our hAQP4 rats can be applied to the analysis of the outcomes of acute-phase therapeutic interventions is still unclear. However, improvement of our model will accumulate basic data to develop investigational new drugs for NMOSD.

## 4. Materials and Methods

### 4.1. Establishment of Humanized-AQP4-Expressing Rats

#### 4.1.1. Plasmid Constructions

Polymerase chain reaction (PCR)-based mutagenesis was performed to introduce V53L, S62T, N64K, T120A, and T149M mutations (Figure S2) for creating the “humanized” rat AQP4 M23 isoform. All products were inserted into the pGEM-T vector (Promega, Madison, WI, USA) for sequencing. cDNAs encoding human, rat, and humanized rat AQP4 M23 and human AQP4 M1 were inserted into pEBMulti-Puro (FUJIFILM Wako Pure Chemical Corporation, Osaka, Japan).

#### 4.1.2. Cell Culture and Transfection

CHO-K1 cells (RCB0285) obtained from RIKEN BRC (Tsukuba, Japan) were maintained in Ham’s F12 medium supplemented with 10% fetal bovine serum, 50 units/mL penicillin, and 50 μg/mL streptomycin at 37 °C in a 5% CO_2_ incubator.

For ELISA, CHO-K1 cells were seeded onto 60 mm dishes at a density of 1 × 10^6^ cells/dish and transfected with each plasmid using Lipofectamine LTX with Plus Reagents (Thermo Fisher Scientific, Waltham, MA, USA) according to the manufacturer’s instructions. Forty-eight hours after transfection, the cells were selected with 10 μg/mL puromycin (InvivoGen, San Diego, CA, USA).

#### 4.1.3. ELISA

Specific binding of monoclonal antibodies and patient-derived IgGs to AQP4 was evaluated with ELISA, as previously described [62], using CHO cells expressing human AQP4 M1, human AQP4 M23, rat AQP4 M23, or humanized rat AQP4 M23. Cells were amplified and seeded onto 96-well plates at a density of 1 × 10^5^ cells/well and fixed with 4% paraformaldehyde (PFA) at 4 °C overnight. Fixed cells were washed with phosphate-buffered saline (PBS) and blocked with 40% Block Ace (KAC Co., Ltd., Kyoto, Japan) in PBS at room temperature for 1 h. After blocking, cells were incubated with various concentrations of human-specific monoclonal antibodies against the ECDs of AQP4, C9401, D12092, or D15107 [34,62], or patient-derived IgGs in 40% Block Ace/PBS at 4 °C overnight, washed three times with PBS, and incubated with HRP-conjugated anti-mouse IgG (1:8000, Sigma-Aldrich, St. Louis, MO, USA) for monoclonal antibodies or HRP-conjugated anti-human IgG (1:20,000, abcam, Cambridge, UK) for patient-derived IgGs in 10% Block Ace/PBS for 1 h. After washing five times with PBS, signals were visualized by incubation with 50 µL of 3, 3’, 5, 5’ tetramethylbenzidine (Sigma-Aldrich, St. Louis, MO, USA) for 30 min and added with 50 µL of Stop Reagent (Sigma-Aldrich, St. Louis, MO, USA). Absorbance at 450 nm was measured with SpectraMax i3x (Molecular Devices, Sunnyvale, CA, USA). As patient-derived IgGs showed nonspecific binding, which was not negligible, the absorbance of CHO cells transfected with an empty vector was also measured, as described above, and specific binding of patient-derived IgGs to AQP4 was calculated.

All three human-specific monoclonal antibodies bound to humanized rat AQP4 M23 with affinities comparable to those for human AQP4 M23 (Figure S2c–e), showing that rat AQP4 was certainly humanized by the introduced mutations (Figure S2a). Thus, genome editing was used to generate hAQP4 rats. 

#### 4.1.4. Animals

LEW/CrlCrlj rats for donor embryos were obtained from The Jackson Laboratory Japan, Inc. (Kanagawa, Japan). Iar: Wistar-Imamichi rats for transplant recipients of genome-edited zygotes were obtained from the Institute for Animal Reproduction (Ibaraki, Japan). All animals were maintained at 23 ± 1.5 °C, 45 ± 15% humidity, and a 12 h light/dark cycle. They were fed a standard pellet diet (MF, Oriental Yeast Co., Tokyo, Japan) and tap water. All experiments in this study were approved by the Animal Research Committee of Osaka University (Permission number: 24-006-042).

#### 4.1.5. Preparation of CRISPR Components and Long Single-Stranded Donor DNAs

The gRNAs were designed using CRISPOR (http://crispor.tefor.net/ (accessed on 23 July 2024)) [63], which predicts unique target sites throughout the rat genome. The target sequences selected for knock-in production were 5′-ATCTTTGTTCTGCTCAGCGT-3′ (left) and 5′-CGGTGAGAGCTCTTCTGTTC-3′ (right) (Figure S2f). These gRNAs were prepared using the Precision gRNA Synthesis Kit (Thermo Fisher Scientific, Waltham, MA, USA). Cas9 mRNA was transcribed in vitro using a mMESSAGE mMACHINE T7 Ultra Kit (Thermo Fisher Scientific) from a linearized plasmid (Addgene plasmid # 72602; http://n2t.net/addgene:72602 (accessed on 23 July 2024); RRID:Addgene_72602) and was purified using a MEGAClear kit (Thermo Fisher Scientific). As the knock-in donor, long single-stranded DNAs (lssDNAs) (Figure S2b,f) were prepared using nicking endonucleases (nickase) as previously reported [64].

#### 4.1.6. Manipulation of Rat Embryos and Electroporation

Pronuclear-stage rat embryos were prepared from 12-week-old females that were superovulated by administering 150 U/kg of pregnant mare serum gonadotropin (PMSG: ASKA Animal Health Co., Tokyo, Japan) and then 75 U/kg of human chorionic gonadotropin (HCG: ASKA Animal Health Co.). After natural mating, pronuclear-stage embryos were obtained from the oviducts of the females and cultured in a modified Krebs–Ringer bicarbonate medium (ARK Resource, Kumamoto, Japan).

In the electroporation (EP), 203 LEW/CrlCrlj embryos 4–6 h after collection were placed into a chamber with 40 μL of serum-free media (Opti-MEM, Thermo Fisher Scientific) containing 400 ng/μL Cas9 mRNA, 200 ng/μL gRNAs, and 20 ng/μL lssDNA. They were electroporated with a 5 mm gap electrode (CUY520P5 Nepa Gene, Chiba, Japan) in a NEPA21 Super Electroporator (Nepa Gene, Chiba, Japan) [65]. Eighty embryos that developed in the two-cell stage after EP of Cas9 mRNA, gRNA, and lssDNA were transferred into the oviducts of four female surrogates anesthetized with isoflurane (DS Pharma Animal Health Co., Ltd., Osaka, Japan). Finally, 11 pups were born, one of which was confirmed to be the knocked-in rat (LEW-Aqp4<em2 Mysi>).

Conventional PCR was used for genotyping using primer sets for WT AQP4 (310 bp), 5′-CATTAACTGGGGTGGCTCAGAGAACCCCCTACCTG-3′ and 5′-GCACAGAAATAAGAACAGAAGAGCTCTCACCGTG-3′; and for humanized AQP4 (277 bp), 5′-CACCATTAACTGGGGTGGCACAGAGAAGCCTTTGC-3′ and 5′-CAGCTCCGATGATGGCCCCGAGGCACTGCGCTGCG-3′ (Figure S2f,g).

### 4.2. Experimental NMOSD Model

#### 4.2.1. Animals

Female Lewis rats (LEW/Crlcrlj) obtained from The Jackson Laboratory Japan, Inc. (Kanagawa, Japan) and hAQP4 rats established as above were housed in the Institute for Animal Experimentation, Tohoku University School of Medicine, under standardized conditions. This experiment was approved by the Ethics Committee of the Tohoku University Graduate School of Medicine Committee on Animal Research (Permission no. 2019MdA-235).

#### 4.2.2. Patients and Antibodies

Patient-derived IgGs were obtained from six patients with AQP4-antibody-positive NMOSD fulfilling the 2015 NMOSD diagnostic criteria [66] in the acute phase. Table 1 shows each patient’s clinical symptoms, EDSS at nadir, EDSS post-treatment, and pre-treatment AQP4-antibody titer during the attack. Control IgGs were obtained from two healthy controls.

IgGs were prepared as follows: patient-derived plasma and control sera were heated at 56 °C in a water bath for 30 min. Each sample was subjected to caprylic acid precipitation [67] to remove unwanted proteins such as fibrinogen. Supernatants were collected, dialyzed with PBS, and filtered through a 0.45 µm filter. Samples were applied to HiTrap™ rProtein A FF columns (GE Healthcare, Chicago, IL, USA), eluted with 0.1 M sodium citrate pH 3.0, and neutralized with 1 M Tris-HCl pH 9.0. Purified IgG underwent buffer exchange with Amicon^®^ Ultra-15 (50k NMWL; Merck, Darmstadt, Germany), and its concentration was adjusted to 10 mg/mL. In this study, IgGs purified from the plasma of six patients (NMO1–NMO6) were referred to as NMO1-IgG, NMO2-IgG, NMO3-IgG, NMO4-IgG, NMO5-IgG, and NMO6-IgG, respectively.

The anti-AQP4 antibody assay for diagnosis and titer determination was performed as previously described [28]. The use of patient plasma for this study was approved by the Ethics Committee of Tohoku University Graduate School of Medicine (2021-1-1035).

#### 4.2.3. Induction of Experimental NMOSD

This study included 48 WT and 69 hAQP4 rats. The number of rats included in each experiment is shown in the figures. Experimental NMOSD was induced as previously described [35]. Briefly, 8–9-week-old female rats were subcutaneously immunized with 200 µL of an encephalitogenic mixture containing 100 µg of MBP from guinea pig brain (Sigma-Aldrich, St. Louis, MO, USA) and complete Freund’s adjuvant (Chondrex Inc., Redmond, WA, USA) containing 100 µg of heat-killed Mycobacterium tuberculosis H37Ra to stimulate the disruption of the blood–brain barrier. The immunization was performed under general anesthesia with isoflurane. After rats developed ascending paresis or body weight loss 9–11 days after immunization, they were injected with 3 mg of D15107, 2/20/40 mg of patient-derived IgG, or 40 mg of control IgG. Their body weights were measured daily, and clinical disability was scored as follows: 0 = no symptoms, 0.5 = dragging the tip of tail, 1.0 = limp tail, 1.5 = limp tail with hindlimb inhibition, 2.0 = hindlimb weakness with gait abnormality, 2.5 = hindlimb weakness with dragging, 3.0 = complete hindlimb paralysis, 3.5 = complete hindlimb paralysis and unable to right posture, 4.0 = forelimb weakness, 4.5 = complete forelimb paralysis or moribund, and 5.0 = dead. Two days after IgG injection, rats were sacrificed with isoflurane overdose and perfused with 4% PFA. All CNS tissues, including the brain, brainstem, optic nerves, and spinal cord, were dissected (the whole spinal cord was divided into 12 equal parts), fixed for another 24–48 h in 4% PFA, and embedded in paraffin for histological analysis.

#### 4.2.4. Histology and Immunohistochemistry

Furthermore, 3 to 4 µm-thick serial sections were cut on a microtome. Each tissue was stained with hematoxylin and eosin (HE) and Klüver–Barrera (KB). Immunohistochemical staining was performed as previously described [35,37] using Histofine^®^ Simple Stain (Nichirei Bioscience, Tokyo, Japan) immunostaining systems. The detailed protocol is provided in the Supplementary File (Table S2).

#### 4.2.5. Histopathological Analyses

To quantify the AQP4 loss lesion area, 12 spinal cord sections (one section in each of the 12 parts) in each rat were stained for AQP4 and analyzed. The percentage of AQP4 loss was calculated for each section by dividing the AQP4 loss area by the whole section area using Fiji [68].

Lesion classification and quantitation of MPO-, CD68-, and CD3-positive cells were performed using NDP.view 2.9.29 (Hamamatsu Photonics, Hamamatsu, Japan). Three spinal cord sections with the largest AQP4 loss lesions were picked per rat and used for evaluation. The lesions were classified as previously described [36] using serial sections stained for AQP4, GFAP, C5b-9, KB, and neurofilament (NF). The serial sections stained for AQP4, MPO, CD68, and CD3 were used to count positive cells. The density of each positive cell population was determined by counting the number of positive cells in four standardized microscopic fields of 50 µm square per section that were randomly placed in the AQP4 loss lesions and calculating the average cell density in 1 mm square for each section.

#### 4.2.6. Statistical Analysis

Welch’s *t*-test, Wilcoxon test, or Dunnett’s T3 was used to compare the percentage of AQP4 loss in the spinal cord sections and the density of MPO-, CD68-, and CD3-positive cells in the lesions between the WT and hAQP4 rats. Welch’s analysis of variance was used to compare the disability score, percentage of AQP4 loss in spinal cord sections, percentage of type 1 lesion, and density of MPO-, CD68-, and CD3-positive cells in the lesions among the six NMO-IgGs. Spearman’s rank correlation coefficient was used to assess the relationship between antibody titer and lesion size. All statistical analyses were performed with GraphPad Prism 8.4.3, and *p* < 0.05 was considered significant. Significance is indicated as * *p* < 0.05, ** *p* < 0.01, *** *p* < 0.001, and **** *p* < 0.0001.

## 5. Conclusions

The hAQP4 rat model we developed with gene-editing technology can successfully reproduce the pathological changes induced by human AQP4-specific antibodies, which were not observed in conventional WT rat models. Furthermore, NMOSD patient-derived IgGs can induce NMOSD-like lesions in hAQP4 rats comparable to human pathology. The comparison of hAQP4 and WT rat lesions suggests that a large proportion of anti-AQP4 antibodies derived from patients with NMOSD presumably bind to the AQP4 domains shared by both human and rat AQP4 ECDs in AQP4 OAP. However, considering different varieties of the binding sites, affinities, and pathogenicities of anti-AQP4 antibodies in NMOSD patients, hAQP4 NMOSD rats may be superior to WT rats in reproducing human NMOSD pathology. With improvements in the experimental protocol, our hAQP4 rat model is expected to make an important contribution to the research for NMOSD pathophysiology and the development of novel treatments.

## Figures and Tables

**Table 1:** Patients’ clinical profiles and anti-AQP4 titers.

Patient	NMO1	NMO2	NMO3	NMO4	NMO5	NMO6
Age	64	62	52	28	49	59
Sex	F	F	F	F	F	M
Clinical phenotype	C	ON	ON	ON + C	APS + My	ON + APS + My
Relapse or first attack	Relapse	Relapse	Relapse	First attack	First attack	First attack
EDSS on nadir	7.5	6	5	4	5	5
EDSS after treatment	6	5	5	1.5	2.5	3
Anti-AQP4 titer of serum at attack	1:524,288	1:32,768	1:131,072	1:4096	1:1024	1:1024
Anti-AQP4 titer of purified-IgG	1:131,072	1:262,144	1:32,768	1:4096	1:1024	1:256

Abbreviations: APS, area postrema syndrome; C, cerebral lesion; EDSS, expanded disability status scale; My, myelitis; ON, optic neuritis.

## Data Availability

The original contributions presented in this study are included in the article/Supplementary Material, further inquiries can be directed to the corresponding authors.

## Supplementary Materials

The following supporting information can be downloaded at: https://www.mdpi.com/article/10.3390/ijms25158169/s1.
