# Supplementary material for: Humanized-Aquaporin-4-Expressing Rat Created by Gene-Editing Technology and Its Use to Clarify the Pathology of Neuromyelitis Optica Spectrum Disorder
Source: Int J Mol Sci. 2024 Jul 26;25(15):8169. doi: 10.3390/ijms25158169 (PMC11311328; doi:10.3390/ijms25158169)
Supplement: Supplementary file 1 [file ijms-25-08169-s001.zip › Supplementary Figures.pdf]

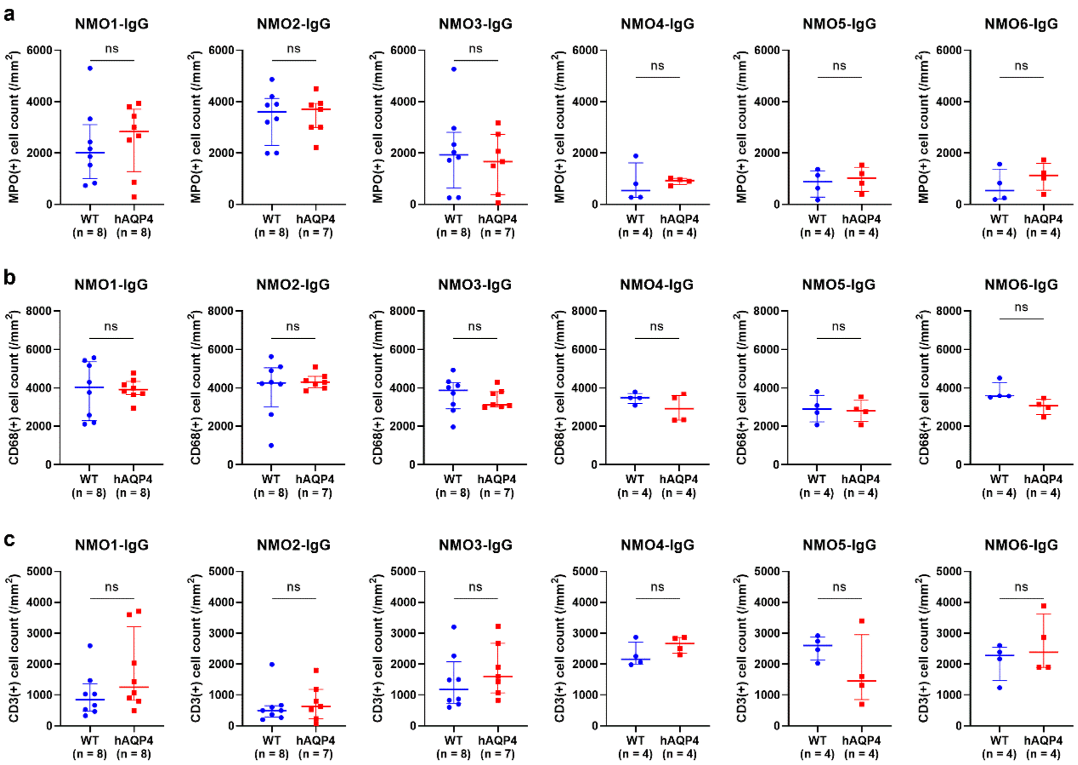

Figure S1. NMOSD patient-derived IgGs induced spinal cord lesions in both WT and hAQP4 rats without significant difference. (a–c) Comparison of complement activation and inflammatory cells in the spinal cord lesions between WT (blue) and hAQP4 (red) rats. Densities of the MPO- (a), CD68- (b), and CD3-positive (c) cells infiltrating the AQP4 loss lesions are shown. Values are median ± IQR with individual points. Statistical analysis was performed using Welch's t-test with GraphPad Prism 8.4.3, and significance is indicated as *p < 0.05, **p < 0.01, ***p < 0.001, and ****p < 0.0001.

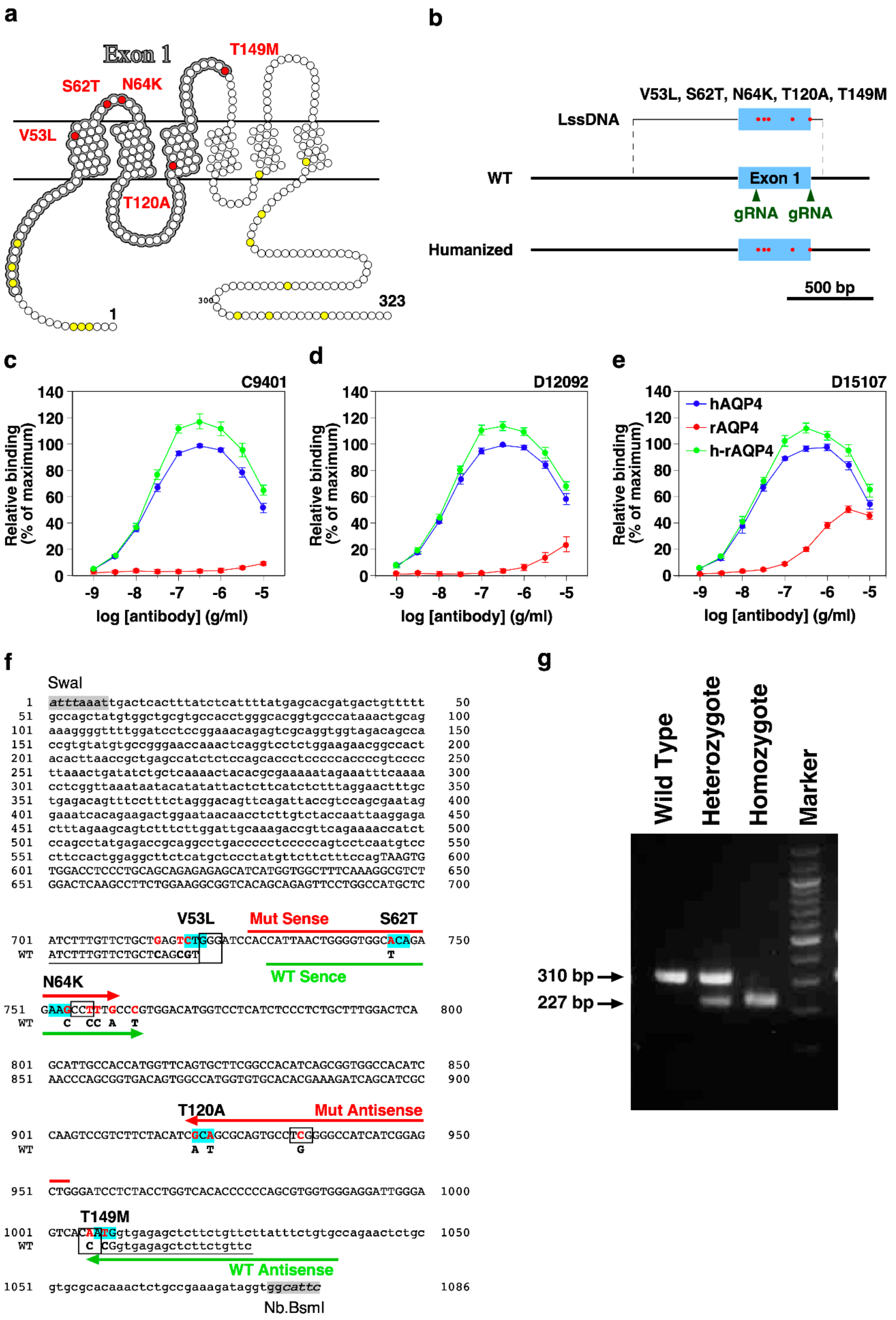
a
Exon 1
T149M
S62T
N64K
V53L
T120A
1
300
323
b
V53L, S62T, N64K, T120A, T149M
LssDNA
WT
Exon 1
gRNA
gRNA
Humanized
500 bp
c
C9401
Relative binding (% of maximum)
log [antibody] (g/ml)
d
D12092
Relative binding (% of maximum)
log [antibody] (g/ml)
e
D15107
hAQP4
rAQP4
h-rAQP4
Relative binding (% of maximum)
log [antibody] (g/ml)
f
SwaI
1 atttaaattgactcactttatctcattttatgagcacgatgactgttttt 50
51 gccagctatgtggctgcgtgccacctgggcacggtgcccataaactgcag 100
101 aaaggggttttggatcctccggaaacagagtcgcaggtggtagacagcca 150
151 ccgtgtatgtgccgggaaccaaactcaggtcctctggaagaacggccact 200
201 acacttaaccgctgagccatctctccagcaccctcccccaccccgtcccc 250
251 ttaaactgatatctgctcaaaactacacgcgaaaaatagaaatttcaaaa 300
301 cctcggttaaataatacatatattactcttcatctctttaggaactttgc 350
351 tgagacagtttcctttctagggacagttcagattaccgtccagcgaatag 400
401 gaaatcacagaagactggaataacaacctcttgtctaccaattaaggaga 450
451 ctttagaagcagtctttcttggattgcaaagaccgttcagaaaaccatct 500
501 ccagcctatgagaccgcaggcctgacccccctcccccagtcctcaatgtcc 550
551 cttccactggaggcttctcatgctccctatgttcttctttccagTAAGTG 600
601 TGGACCTCCCTGCAGCAGAGAGAGCATCATGGTGGCTTTCAAAGGCGTCT 650
651 GGACTCAAGCCTTCTGGAAGGCGGTCACAGCAGAGTTCCTGGCCATGCTC 700
V53L
Mut Sense
S62T
701 ATCTTTGTTCTGCTGAGTCTGGGATCCACCATTAACTGGGGTGGCACAGA 750
WT ATCTTTGTTCTGCTCAGCGT T
WT Sence
N64K
751 GAAGCCTTTGCCCGTGGACATGGTCCTCATCTCCCTCTGCTTTGGACTCA 800
WT C CC A T
801 GCATTGCCACCATGGTTCAGTGCTTCGGCCACATCAGCGGTGGCCACATC 850
851 AACCCAGCGGTGACAGTGGCCATGGTGTGCACACGAAAGATCAGCATCGC 900
T120A
Mut Antisense
901 CAAGTCCGTCTTCTACATCGCAGCGCAGTGCCTCGGGGCCATCATCGGAG 950
WT A T G
951 CTGGGATCCTCTACCTGGTCACACCCCCCAGCGTGGTGGGAGGATTGGGA 1000
T149M
1001 GTCACAATGgtgagagctcttctgttcttatttctgtgccagaactctgc 1050
WT C CGgtgagagctcttctgttc
WT Antisense
1051 gtgcgcacaaactctgccgaaagataggtggcattc 1086
Nb.BsmI
g
Wild Type
Heterozygote
Homozygote
Marker
310 bp
227 bp

Figure S2. Establishment of hAQP4 rats. (a) Schematic illustration of humanized rat AQP4 M1. Five amino acids in rat AQP4 encoded by exon 1 were substituted with those in human AQP4 (V53L, S62T, M64K, T102A, and T149M). Amino acids different between rat and human AQP4 are colored. Those changed and unchanged in the humanized rat AQP4 are indicated in red and yellow, respectively. The range of the amino acid sequence encoded by exon 1 is indicated in gray. (b) Schematic illustration of the strategy for establishing hAQP4 rats. Exon 1 is indicated with a blue box. Locations of introduced mutations for humanizing rat AQP4 are indicated with red dots. The gRNA target sites are indicated with green arrowheads. (c–e) The dose-dependent binding of human-specific antibodies [35, 61] against the extracellular domains of AQP4. Binding of C9401 (c), D12092 (d), and D15107 (e) against human (blue), rat (red), and humanized rat (green) AQP4 M23 expressed in CHO-K1 cells was examined by ELISA and shown as % of the maximal binding to human AQP4 M23. Values are mean ± SEM of six independent experiments. (f) Sequence of lssDNA used to establish the hAQP4 rats. Sequences of exon 1 and introns are written in uppercase and lowercase letters, respectively. Nucleotides substituted in the lssDNA are indicated in red. Codons that changed from rat to human are highlighted in blue. Sequences in wild-type rat *Aqp4* recognized by gRNAs are underlined. The positions of potential protospacer adjacent motifs are indicated with boxes. Genotyping primers detecting wild-type and humanized rat *Aqp4* alleles are indicated with green and red arrows, respectively. (g) A representative electrophoretic pattern of polymerase chain reaction products for genotyping.
