# Supplementary material for: Humanized-Aquaporin-4-Expressing Rat Created by Gene-Editing Technology and Its Use to Clarify the Pathology of Neuromyelitis Optica Spectrum Disorder
Source: Int J Mol Sci. 2024 Jul 26;25(15):8169. doi: 10.3390/ijms25158169 (PMC11311328; doi:10.3390/ijms25158169)
Supplement: Supplementary file 1 [file ijms-25-08169-s001.zip › Supplementary Tables.pdf]

Table S1. Clinical and pathological findings of the hAQP4 rats.

transferred IgG	NMO1-IgG (n = 8)	NMO2-IgG (n = 8)	NMO3-IgG (n = 8)	NMO4-IgG (n = 4)	NMO5-IgG (n = 4)	NMO6-IgG (n = 4)	p†
Disability score on day 2	2.94±0.36	2.75±0.41	2.75±0.28	3.25±0.25	3.13±0.59	2.75±0.63	0.85
AQP4 loss (%)	3.17±0.78	9.75±2.78	1.70±1.10	0.52±0.19	0.40±0.12	0.18±0.05	0.0086**
Type1 lesion (%)	90.63±6.09	98.85±0.95	94.74±3.14	88.40±5.37	87.92±4.36	94.29±4.04	0.18
MPO-positive cells (/mm^2)	2561±471	3459±289	1653±431	908±129	992±243	1100±276	0.0002***
CD68-positive cells (/mm^2)	3931±195	4344±157	3215±196	2942±363	2803±300	3010±208	0.0037**
CD3-positive cells (/mm^2)	1756±445	750±223	1818±325	2625±136	1753±580	2637±474	0.0014**
Cerebral lesion	4/8	7/8	5/8	4/4	3/4	3/4	
Optic nerve lesion	3/8	2/8	0/8	1/4	1/4	2/4	
Midbrain lesion	4/8	5/8	2/8	1/4	1/3	1/4	
Pons lesion	6/8	6/8	5/8	2/4	2/4	2/4	
Cerebellar lesion	4/8	3/8	2/8	2/4	2/4	1/4	
Medulla lesion	7/8	7/8	7/8	4/4	2/4	3/4	
Spinal cord lesion	8/8	7/8	7/8	4/4	4/4	4/4	

† Welch's analysis of variance was used to compare the disability score, percentage of AQP4 loss in the spinal cord sections, percentage of type 1 lesion and density of MPO-positive, CD68-positive, and CD3-positive cells in the lesions among the 6 NMO-IgGs. Statistical analysis was performed with GraphPad Prism 8.4.3, and significance is indicated as $*p < 0.05$, $**p < 0.01$, $***p < 0.001$, and $****p < 0.0001$.

Table S2. Primary antibodies and conditions for immunohistochemistry*.

Antibody	Origin	Clonality	Dilution	Antigen retrieval	Source	No.
**AQP4**	rabbit	polyclonal	1:200	100°C, 5 min	Santa Cruz Biotechnology, Dallas, TX, USA	SC-20812
**GFAP**	rabbit	polyclonal	1:250	100°C, 5 min	Proteintech, Chicago, IL, USA	16825-1-AP
**MBP**	rabbit	polyclonal	1:500	-	Dako, Glostrup, Denmark	A0623
**MAG**	rabbit	polyclonal	1:50	100°C, 5 min	Sigma-Aldrich, St. Louis, MO, USA	HPA012499
**NF**	mouse	monoclonal	1:1000	100°C, 5 min	Calbiochem, San Diego, CA, USA	NE1022
**rat C5b-9**	mouse	monoclonal	1:500	0.05% proteinase type XXIV, 37°C, 10 min	Hycult Biotech, Uden, Netherlands	HM3033
**MPO**	rabbit	monoclonal	1:1000	-	Abcam, Cambridge, UK	ab208670
**CD68**	rabbit	polyclonal	1:500	125°C, 5 min	Abcam, Cambridge, UK	ab125212
**CD3**	rabbit	polyclonal	1:100	125°C, 5 min	Abcam, Cambridge, UK	ab5690
**CD20**	rabbit	polyclonal	1:400	125°C, 5 min	Bioss, Boston, MA, USA	bs-0080R

* Immunohistochemical examinations were done as follows,
the sections were deparaffinized in xylene, rehydrated in ethanol, and rinsed with PBS. Antigen retrieval was performed by heating samples in Diva Decloaker heat retrieval solution (Biocare Medical, Pacheco, CA, USA) within a Decloaking Chamber (model DC2002; Biocare Medical, Pacheco, CA, USA) or heating for 10 min at 37 °C in 0.05% proteinase type XXIV (Sigma-Aldrich, St. Louis, MO, USA) as needed. After blocking nonspecific binding with 10% goat serum for 15 min at room temperature, the slides were covered and incubated with primary antibodies at 4 °C overnight. The primary antibodies and specific conditions for antigen retrieval are shown in Table S2. After incubation with primary antibodies, the sections were washed in PBS and incubated in 30% methanol/PBS containing 1% hydrogen peroxide for 20 min to block endogenous peroxidase. The sections were then washed in PBS, incubated with the corresponding secondary antibody for 40 min at room temperature, and then washed in PBS. For staining, 3,3′-diaminobenzene tetrahydrochloride (DAB; brown) was used for the horseradish peroxidase. The sections were counterstained with hematoxylin (blue), dehydrated, and mounted in Entellan™ new (Merck, Darmstadt, Germany).
